# Routine HIV clinic visit adherence in the African Cohort Study

**DOI:** 10.1186/s12981-021-00425-0

**Published:** 2022-01-07

**Authors:** Nicole Dear, Allahna Esber, Michael Iroezindu, Emmanuel Bahemana, Hannah Kibuuka, Jonah Maswai, John Owuoth, Christina S. Polyak, Julie A. Ake, Trevor A. Crowell, the AFRICOS Study Group, Danielle Bartolanzo, Alexus Reynolds, Katherine Song, Mark Milazzo, Leilani Francisco, Shauna Mankiewicz, Steven Schech, Alexandra Golway, Badryah Omar, Tsedal Mebrahtu, Elizabeth Lee, Kimberly Bohince, Ajay Parikh, Jaclyn Hern, Emma Duff, Kara Lombardi, Michelle Imbach, Leigh Anne Eller, Hannah Kibuuka, Michael Semwogerere, Prossy Naluyima, Godfrey Zziwa, Allan Tindikahwa, Hilda Mutebe, Cate Kafeero, Enos Baghendaghe, William Lwebuge, Freddie Ssentogo, Hellen Birungi, Josephine Tegamanyi, Paul Wangiri, Christine Nabanoba, Phiona Namulondo, Richard Tumusiime, Ezra Musingye, Christina Nanteza, Joseph Wandege, Michael Waiswa, Evelyn Najjuma, Olive Maggaga, Isaac Kato Kenoly, Barbara Mukanza, Jonah Maswai, Rither Langat, Aaron Ngeno, Lucy Korir, Raphael Langat, Francis Opiyo, Alex Kasembeli, Christopher Ochieng, Japhet Towett, Jane Kimetto, Brighton Omondi, Mary Leelgo, Michael Obonyo, Linner Rotich, Enock Tonui, Ella Chelangat, Joan Kapkiai, Salome Wangare, Zeddy Bett Kesi, Janet Ngeno, Edwin Langat, Kennedy Labosso, Joshua Rotich, Leonard Cheruiyot, Enock Changwony, Mike Bii, Ezekiel Chumba, Susan Ontango, Danson Gitonga, Samuel Kiprotich, Bornes Ngtech, Grace Engoke, Irene Metet, Alice Airo, Ignatius Kiptoo, John Owuoth, Valentine Sing’oei, Winne Rehema, Solomon Otieno, Celine Ogari, Elkanah Modi, Oscar Adimo, Charles Okwaro, Christine Lando, Margaret Onyango, Iddah Aoko, Kennedy Obambo, Joseph Meyo, George Suja, Michael Iroezindu, Yakubu Adamu, Nnamdi Azuakola, Mfreke Asuquo, Abdulwasiu Bolaji Tiamiyu, Afoke Kokogho, Samirah Sani Mohammed, Ifeanyi Okoye, Sunday Odeyemi, Aminu Suleiman, Lawrence Umejo, Onome Enas, Miriam Mbachu, Ijeoma Chigbu-Ukaegbu, Wilson Adai, Felicia Anayochukwu Odo, Rabi Abdu, Rosemary Akiga, Helen Nwandu, CHisara Okolo, Ndubuisis Okeke, Zahra Parker, Asogwa Ugochukwu Linus, Concilia Amaka Agbaim, Tunde Adegbite, Nkenchiere Harrison, Adewale Adelakun, Ekeocha Chioma, Victoria Idi, Rachel Eluwa, Jumoke Nwalozie, Igiri Faith, Blessing Okanigbuan, Achugwo Emmanuel, Nkiru Nnadi, Ndubuisi Rosemary, Uzoegwu Amaka Natalie, Obende Theresa Owanza, Falaju Idowu Francis, Jacintal Elemere, Obilor Ifeoma Lauretta, Edward Akinwale, Inalegwu Ochai, Lucas Maganga, Emmanuel Bahemana, Samoel Khamadi, John Njegite, Connie Lueer, Abisai Kisinda, Jaquiline Mwamwaja, Faraja Mbwayu, Gloria David, Mtasi Mwaipopo, Reginald Gervas, Doroth Mkondoo, Nancy Somi, Paschal Kiliba, Gwamaka Mwaisanga, Johnisius Msigwa, Hawa Mfumbulwa, Peter Edwin, Willyhelmina Olomi

**Affiliations:** 1 U.S. Military HIV Research Program, Walter Reed Army Institute of Research, Silver Spring, MD USA; 2 Henry M. Jackson Foundation for the Advancement of Military Medicine, 6720A Rockledge Dr, Suite 400, Bethesda, MD 20817 USA; 3 HJF Medical Research International, Abuja, Nigeria; 4 HJF Medical Research International, Mbeya, Tanzania; 5 Makerere University Walter Reed Project, Kampala, Uganda; 6 U.S. Army Medical Research Directorate - Africa, Kericho, Kenya; 7 HJF Medical Research International, Kisumu, Kenya

**Keywords:** HIV, East Africa, West Africa, Clinic visits, Patient engagement, Care retention

## Abstract

**Background:**

Retention in clinical care is important for people living with HIV (PLWH). Evidence suggests that missed clinic visits are associated with interruptions in antiretroviral therapy (ART), lower CD4 counts, virologic failure, and overlooked coinfections. We identified factors associated with missed routine clinic visits in the African Cohort Study (AFRICOS).

**Methods:**

In 2013, AFRICOS began enrolling people with and without HIV in Uganda, Kenya, Tanzania, and Nigeria. At enrollment and every 6 months thereafter, sociodemographic questionnaires are administered and clinical outcomes assessed. Missed clinic visits were measured as the self-reported number of clinic visits missed in the past 6 months and dichotomized into none or one or more visits missed. Logistic regression with generalized estimating equations was used to estimate odds ratios (ORs) and 95% confidence intervals (CIs) for associations between risk factors and missed visits.

**Results:**

Between January 2013 and March 2020, 2937 PLWH were enrolled, of whom 2807 (95.6%) had initiated ART and 2771 had complete data available for analyses. Compared to PLWH 50+, missed clinic visits were more common among those 18–29 years (aOR 2.33, 95% CI 1.65–3.29), 30–39 years (aOR 1.59, 95% CI 1.19–2.13), and 40–49 years (aOR 1.42, 95% CI 1.07–1.89). As compared to PLWH on ART for < 2 years, those on ART for 4+ years were less likely to have missed clinic visits (aOR 0.72, 95% CI 0.55–0.95). Missed clinic visits were associated with alcohol use (aOR 1.34, 95% CI 1.05–1.70), a history of incarceration (aOR 1.42, 95% CI 1.07–1.88), depression (aOR 1.47, 95% CI 1.13–1.91), and viral non-suppression (aOR 2.50, 95% CI 2.00–3.12). As compared to PLWH who did not miss any ART in the past month, missed clinic visits were more common among those who missed 1–2 days (aOR 2.09, 95% CI 1.65–2.64) and 3+ days of ART (aOR 7.06, 95% CI 5.43–9.19).

**Conclusions:**

Inconsistent clinic attendance is associated with worsened HIV-related outcomes. Strategies to improve visit adherence are especially needed for young PLWH and those with depression.

**Supplementary Information:**

The online version contains supplementary material available at 10.1186/s12981-021-00425-0.

## Background

Retention in clinical care, including routine clinic visit attendance, is important for all people living with HIV (PLWH), whether feeling unwell or thriving with HIV managed as a chronic disease. Evidence suggests that missed routine clinic visits, often considered short treatment interruptions, are associated with interruptions in antiretroviral therapy (ART), lower CD4 counts, virologic failure, increased risk for drug resistance, and overlooked coinfections [1–11]. Conversely, consistent clinic visit attendance provides opportunities for healthcare providers to evaluate for opportunistic infections, encourage medication adherence and health promoting behaviors, manage side effects, detect drug toxicities, and modify ART when needed [12], with potential population level reductions in HIV transmission [9, 13].

Despite the importance of retention in HIV clinical care, data suggest that missing clinic visits is common, particularly in the first year after treatment initiation. A retrospective cohort study in Johannesburg, South Africa found that out of an anticipated five ART pickup and three medical visits, 26% of PLWH missed one medical or ART visit, 7% missed two visits, and 2% missed three or more visits during the first 6 months on ART [1]. Another retrospective cohort study of PLWH receiving care at a regional referral hospital in Uganda found that over a 20 month study period, 59% of PLWH in care had ever missed a scheduled clinic visit [2].

Complex socio-structural, behavioral, and economic factors contribute to missing clinic visits. Evidence from a clinical care setting in Uganda suggests that socio-structural barriers contribute to clinic visit nonadherence, including having to travel far to the clinic, HIV-related stigma, forgetting the appointment or not being able to take time off work, experiencing ART side effects, having an adequate drug supply, and food insecurity [2]. A cross-sectional study in Johannesburg, South Africa found that older and more educated PLWH were more likely to miss clinic visits, and the most commonly reported reasons for missing clinic visits included transportation costs, being away for work or personal travel, having forgotten the appointment, and not feeling well [14].

In April 2021, the World Health Organization (WHO) published updates to guidelines initially released in 2016 in support of a differentiated service delivery approach [15]. While frequent clinic attendance may be important for certain individuals at certain times, the WHO supports less frequent clinical visits for stable PLWH to reduce the burden of care for both clients and health systems [15], and which has been found to be associated with improved engagement including decreased lateness, fewer medication interruptions, and decreased loss to follow-up [16].

While attrition from care has been well documented in low resource settings, including in sub-Saharan Africa [12, 17, 18], intermittent adherence to clinic visits is not as well described. We longitudinally assessed potential risk factors for missed clinic visits in four African countries with a high burden of HIV.

## Methods

### Study population

Since 2013, the African Cohort Study (AFRICOS) has prospectively enrolled people with and without HIV, in an approximate 5:1 ratio, at 12 President’s Emergency Plan for AIDS Relief (PEPFAR) supported facilities across five established US Military HIV Research Program sites: Uganda; South Rift Valley, Kenya; Kisumu, Kenya; Tanzania; and Nigeria [19, 20]. AFRICOS longitudinally assesses the impact of clinical practices and biological and socio-behavioral factors on HIV infection and disease progression in an African context. PLWH were eligible for enrollment if they were 18 years or older and receiving HIV care at the enrolling PEPFAR clinic. Beginning in January 2020 eligibility expanded to include individuals 15–17 years. Individuals who were pregnant and those with any significant condition that would interfere with study procedures were not eligible for enrollment.

The study was approved by institutional review boards of the Walter Reed Army Institute of Research, Makerere University School of Public Health, Kenya Medical Research Institute, Tanzania National Institute of Medical Research, and the Nigerian Ministry of Defence. All participants provided written informed consent.

### Data collection and measures

At enrollment and every 6 months thereafter, participants received a clinical assessment and a socio-behavioral questionnaire was administered by trained study staff. Self-reported demographic and socio-behavioral variables included age; sex; marital status; education and employment status. To assess alcohol and drug use, participants were asked the following at each visit: “Do you consume alcohol?” and “Have you used recreational drugs, such as inhalants, consumables, or injectables?” with “yes”, “no” or “no response” response options. Self-reported structural factors included food security, defined as having enough food to eat in the past 12 months [21]; lifetime history of incarceration, defined as having ever spent time in prison; time and distance to clinic; and satisfaction with clinic waiting time.

Self-reported HIV-specific variables included HIV-related stigma associated events, defined as experiencing any of the following: social isolation, physical violence, broken family relationships [22]; status disclosure, defined as disclosure to any of the following individuals: spouse or partner, parent, sibling, children, grandparents, extended family members, friend, roommate, church members; and ART adherence, based on the self-reported number of days of ART missed in the past 30 days.

Depression was assessed at enrollment and subsequent visits using the 20-item Center for Epidemiological Studies-Depression (CES-D) scale, and dichotomized, with a score of 16 or greater suggestive of depression [23, 24]. Other clinical variables included ART status, year of ART initiation, and duration on ART, ascertained from medical record review; tuberculosis (TB) coinfection, diagnosed by a positive GeneXpert, mycobacterial smear or mycobacterial culture result; CD4 count; and viral load [19].

Study visits were generally scheduled to coincide with routine clinic visits. Routine clinical care and treatment for HIV and acute illnesses is performed by clinic staff per local standard of care. AFRICOS study staff then record the results of the routine clinical care (ex. lab results, ART prescribed) and conduct the remainder of study procedures.

Missed routine HIV clinic visits were based on self-report and measured as the number of clinic visits missed in the past 6 months. The expected number of clinic visits was based on the self-reported frequency of follow-up visits in a 6-month period. Missed clinic visits were classified two ways for analyses: (1) dichotomized into no missed visits or one or more missed visits regardless of frequency of expected visits; (2) the proportion of expected clinic visits missed was calculated by dividing the number of visits missed by the total number of clinic visits that were expected in the past 6 months and categorized into four levels as follows: 0% missed, 1–49% missed, 50–99% missed, 100% missed.

Participants were asked to provide a reason if they reported missing visits. Participants could provide more than one response and/or specify a free text response. Text responses were retrospectively recoded into an existing option or coded as a new option.

### Statistical analyses

These analyses included PLWH on ART and were conducted to address, in part, the following secondary objective of the AFRICOS protocol: describe adherence to HIV care and evaluate for factors associated with HIV clinic visit adherence. Characteristics were described for PLWH on ART attending routine clinic visits at their first study visit on ART. p-values were calculated using Pearson’s chi-squared and Wilcoxon rank-sum tests.

The self-reported frequency of expected clinic visits over time and the proportion of participants missing one or more clinic visits by frequency of routine clinic visits were analyzed descriptively. Reasons for missing clinic visits were also analyzed descriptively.

Logistic regression with generalized estimating equations, clustered by participant to account for repeated measures, was used to estimate unadjusted and adjusted odds ratios (ORs) and 95% confidence intervals (CIs) for factors potentially associated with missed HIV clinic visits in the previous 6-month period. All potential risk factors specified a priori were included in the adjusted model regardless of significance of the association. We tested for multicollinearity using the variable inflation factor (VIF); all variables included in the adjusted model had a VIF < 2. Analyses were restricted to complete cases only.

We ran a sensitivity analysis using multinomial logistic regression to assess factors associated with the proportion of expected visits missed in the previous 6-month period, comparing the following categories of proportions of expected visits missed to participants who missed zero percent of their expected visits: 1–49% missed, 50–99% missed, 100% missed. To account for multiple visits by a participant, analyses were clustered on participant and included a robust variance estimator. Like the main model, all potential predictors were included in the fully adjusted model.

Analyses were performed in SAS version 9.3 (SAS Institute, Cary, North Carolina) and Stata version 16.0 (StataCorp, College Station, Texas).

## Results

### Characteristics of the study population

Between January 2013 and March 2020, 3551 participants were enrolled, including 2937 (82.7%) PLWH, of whom 2807 (95.6%) had record of initiating ART at any point before or during follow-up. After restricting to complete cases for all covariates including the outcome of interest, 2771 PLWH on ART were included in further analyses. For the participants included in these analyses, enrollment and follow-up visits occurred between January 2013 and March 2020, with a median follow-up time of 3.6 (interquartile range (IQR) 2.1–4.8) years; 203 (7.3%) participants contributed only one visit.

At the first study visit included in these analyses, the median age of participants was 38.9 years ((IQR 31.9–46.4 years) and 1620 (58.5%) were female (Table 1). The median distance was 8 km (IQR 4–16 km) and the median time to clinic was 50 min (IQR 30–90 min).

**Table 1:** Characteristics of AFRICOS participants living with HIV on ART attending routine HIV clinic visits at first study visit on antiretroviral therapy, by missed visit status

	Total (n = 2771)	No missed visits (n = 2581)	Missed visits (n = 190)	p-value
Age at visit (years)				0.11
18–29	528 (19.1%)	484 (18.8%)	44 (23.2%)	
30–39	979 (35.3%)	905 (35.1%)	74 (38.9%)	
40–49	788 (28.4%)	739 (28.6%)	49 (25.8%)	
50+	476 (17.2%)	453 (17.6%)	23 (12.1%)	
Sex				0.10
Male	1151 (41.5%)	1083 (42.0%)	68 (35.8%)	
Female	1620 (58.5%)	1498 (58.0%)	122 (64.2%)	
Program site				** < 0.001**
Kayunga, Uganda	474 (17.1%)	424 (16.4%)	50 (26.3%)	
South Rift Valley, Kenya	987 (35.6%)	922 (35.7%)	65 (34.2%)	
Kisumu West, Kenya	498 (18.0%)	466 (18.1%)	32 (16.8%)	
Mbeya, Tanzania	520 (18.8%)	505 (19.6%)	15 (7.9%)	
Abuja and Lagos Nigeria	292 (10.5%)	264 (10.2%)	28 (14.7%)	
Marital status				0.08
Not married	1203 (43.4%)	1109 (43.0%)	94 (49.5%)	
Married	1568 (56.6%)	1472 (57.0%)	96 (50.5%)	
Education				0.25
None or some primary	907 (32.7%)	836 (32.4%)	71 (37.4%)	
Primary or some secondary	1094 (39.5%)	1029 (39.9%)	65 (34.2%)	
Secondary and above	770 (27.8%)	716 (27.7%)	54 (28.4%)	
Employment status				** < 0.01**
Unemployed	1667 (60.2%)	1570 (60.8%)	97 (51.1%)	
Employed	1104 (39.8%)	1011 (39.2%)	93 (48.9%)	
Alcohol use				** < 0.01**
No	2330 (84.1%)	2185 (84.7%)	145 (76.3%)	
Yes	441 (15.9%)	396 (15.3%)	45 (23.7%)	
Recreational drug use				0.44
No	2706 (97.7%)	2522 (97.7%)	184 (96.8%)	
Yes	65 (2.3%)	59 (2.3%)	6 (3.2%)	
Enough food to eat^a^				0.20
No	934 (33.7%)	878 (34.0%)	56 (29.5%)	
Yes	1837 (66.3%)	1703 (66.0%)	134 (70.5%)	
Ever incarcerated				**0.02**
No	2477 (89.4%)	2317 (89.8%)	160 (84.2%)	
Yes	294 (10.6%)	264 (10.2%)	30 (15.8%)	
Distance to clinic				0.27
< 10 km	1439 (51.9%)	1333 (51.6%)	106 (55.8%)	
10+ km	1332 (48.1%)	1248 (48.4%)	84 (44.2%)	
Time to clinic				0.77
≤ 30 min	1081 (39.0%)	1005 (38.9%)	76 (40.0%)	
> 30 min	1690 (61.0%)	1576 (61.1%)	114 (60.0%)	
Waiting time				0.24
Satisfied	2356 (85.0%)	2200 (85.2%)	156 (82.1%)	
Needs to improve	415 (15.0%)	381 (14.8%)	34 (17.9%)	
Depression^b^				0.19
No	2405 (86.8%)	2246 (87.0%)	159 (83.7%)	
Yes	366 (13.2%)	335 (13.0%)	31 (16.3%)	
TB diagnosis^c^				0.49
No	2690 (97.1%)	2504 (97.0%)	186 (97.9%)	
Yes	81 (2.9%)	77 (3.0%)	4 (2.1%)	
Year started ART				0.65
1999–2005	95 (3.4%)	89 (3.4%)	6 (3.2%)	
2006–2010	731 (26.4%)	683 (26.5%)	48 (25.3%)	
2011–2015	1326 (47.9%)	1227 (47.5%)	99 (52.1%)	
2016–2019	619 (22.3%)	582 (22.5%)	37 (19.5%)	
Duration on ART				0.38
< 2 years	1564 (56.4%)	1460 (56.6%)	104 (54.7%)	
≥ 2 years to < 4 years	348 (12.6%)	318 (12.3%)	30 (15.8%)	
≥ 4 years	859 (31.0%)	803 (31.1%)	56 (29.5%)	
Experienced HIV stigma^d^				0.41
No	2521 (91.0%)	2345 (90.9%)	176 (92.6%)	
Yes	250 (9.0%)	236 (9.1%)	14 (7.4%)	
Disclosed HIV status^e^				0.15
No	450 (16.2%)	412 (16.0%)	38 (20.0%)	
Yes	2321 (83.8%)	2169 (84.0%)	152 (80.0%)	
Missed days of ART in past month				** < 0.001**
No days missed	2344 (84.6%)	2227 (86.3%)	117 (61.6%)	
1–2 days missed	306 (11.0%)	266 (10.3%)	40 (21.1%)	
3+ days missed	121 (4.4%)	88 (3.4%)	33 (17.4%)	
CD4 count				0.38
< 200 cells/mm^3^	420 (15.2%)	387 (15.0%)	33 (17.4%)	
≥ 200 cells/mm^3^	2351 (84.8%)	2194 (85.0%)	157 (82.6%)	
Viral load				** < 0.001**
< 1000 copies/mL	2418 (87.3%)	2269 (87.9%)	149 (78.4%)	
≥ 1000 copies/mL	353 (12.7%)	312 (12.1%)	41 (21.6%)	

Between January 2013 and March 2020, people living with HIV were enrolled at 12 HIV clinics in Kenya, Uganda, Tanzania, and Nigeria. Characteristics are summarized from their first study visit on antiretroviral therapy, which may have been at enrollment into the cohort or later if they were ART-naïve at enrollment. Missed clinic visits were based on self-report and defined as one or more missed clinic visits in the past 6 months. Data are presented as n (column %). P-values were calculated using Pearson’s chi-squared tests and statistically significant p-values (p < 0.05) are shown in bold

^a^Enough food to eat in the past 12 months

^b^Center for Epidemiologic Studies Depression (CES-D) Scale score, dichotomized with a score of 16 or greater suggestive of depression

^c^Tuberculosis (TB) coinfection, diagnosed by a positive GeneXpert, mycobacterial smear or mycobacterial culture result

^d^Participants were defined as experiencing stigma if they had experienced any of the following: social isolation, physical violence, broken family relationships

^e^Disclosure status was defined as disclosure to any of the following individuals: spouse/partner, parent, sibling, children, grandparents, extended family members, friend, roommate, church members

### Frequency of expected clinic visits

**Fig. 1:**
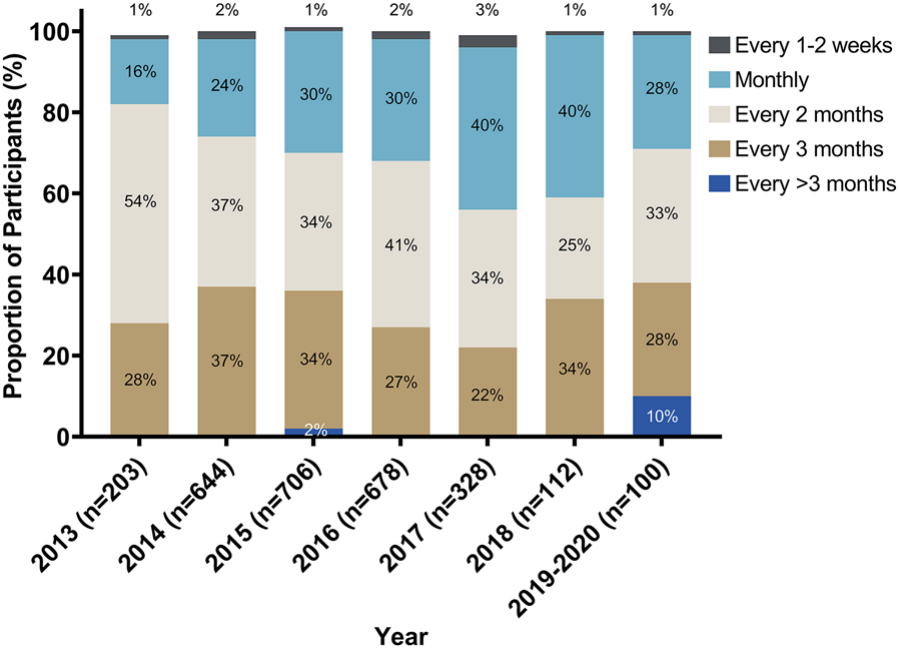
Frequency of expected clinic visits by year; p < 0.001. The p-value was calculated using a Pearson’s chi-squared test. Between January 2013 and March 2020, people living with HIV were enrolled at 12 HIV clinics in Kenya, Uganda, Tanzania, and Nigeria. The frequency of expected clinic visits is from their first study visit on antiretroviral therapy, which may have been at enrollment into the cohort or later if they were ART-naïve at enrollment. The frequency of expected clinic visits describes how often clients reported they were expected to attend routine clinic visits in the 6-month period prior to the study visit and is based on self-report

**Fig. 2:**
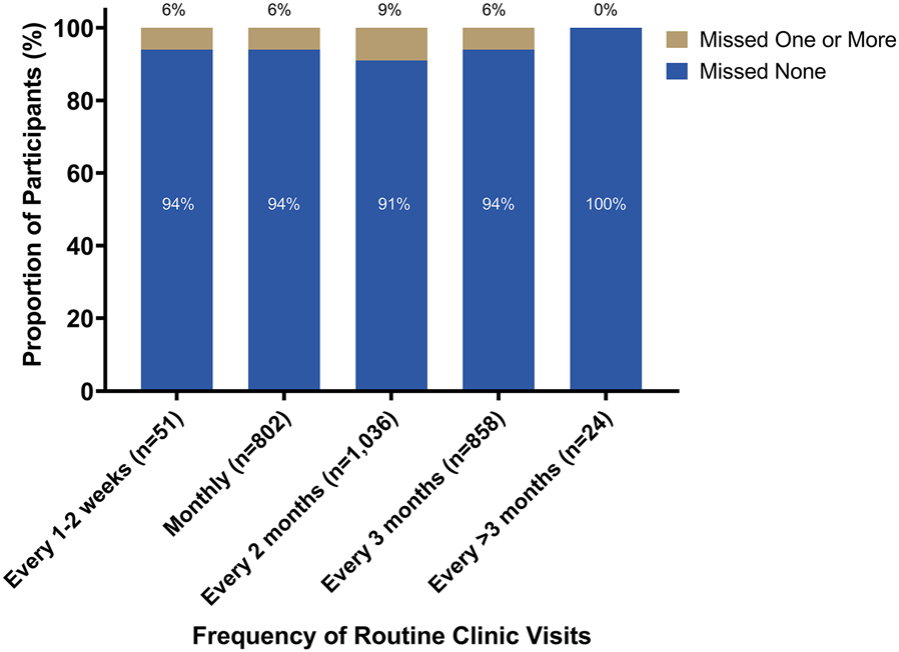
Proportion of participants missing one or more clinic visits by frequency of routine clinic visits; p = 0.02. The p-value was calculated using a Pearson’s chi-squared test. Between January 2013 and March 2020, people living with HIV were enrolled at 12 HIV clinics in Kenya, Uganda, Tanzania, and Nigeria. The frequency of expected clinic visits is from their first study visit on antiretroviral therapy, which may have been at enrollment into the cohort or later if they were ART-naïve at enrollment. Missed HIV clinic visits were based on self-report and measured as the number of clinic visits missed in the past 6 months. The frequency of expected clinic visits describes how often clients reported they were expected to attend routine clinic visits in the 6-month period prior to the study visit and is based on self-report

The self-reported frequency of clinic visits expected in the 6-month period prior to a study visit varied by participant. Figure 1 presents the self-reported frequency of expected clinic visits at the first study visit included by year. There was a shift toward fewer clinical visits over time (p < 0.001). The proportion of participants missing one or more clinic visits was greater among those with clinic visits scheduled for every 3 months or more frequently (p = 0.02; Fig. 2).

### Factors associated with missed visits

As compared to PLWH aged 50 years and older, missed clinic visits were more common among those aged 18–29 years (aOR 2.33, 95% CI 1.65–3.29), 30–39 years (aOR 1.59, 95% CI 1.19–2.13) and 40–49 years (aOR 1.42, 95% CI 1.07–1.89; Table 2). Those in Nigeria had higher odds of missing visits (aOR 1.53, 95% CI 1.04–2.27) and those in Tanzania had lower odds of missing visits (aOR 0.30, 95% CI 0.19–0.47) as compared to participants in Uganda. As compared to PLWH who had been on ART for less than 2 years, those who had been on ART for 4 years or more were less likely to have missed clinic visits (aOR 0.72, 95% CI 0.55–0.95).

**Table 2:** Unadjusted and adjusted logistic regression models comparing factors collected at 6-monthly study visits with missing one or more routine HIV clinic visits in the previous 6-months

	Unadjusted OR (95% CI)	Adjusted OR (95% CI)
Age at visit (years)		
18–29	**3.23 (2.33–4.48)**	**2.33 (1.65–3.29)**
30–39	**2.26 (1.68–3.02)**	**1.59 (1.19–2.13)**
40–49	**1.67 (1.25–2.24)**	**1.42 (1.07–1.89)**
50+	Ref.	–
Sex		
Male	Ref.	–
Female	1.14 (0.94–1.38)	1.17 (0.95–1.44)
Program site		
Kayunga, Uganda	Ref.	–
South Rift Valley, Kenya	**0.62 (0.48–0.80)**	0.78 (0.56–1.10)
Kisumu West, Kenya	**0.57 (0.44–0.75)**	0.69 (0.48–1.01)
Mbeya, Tanzania	**0.33 (0.23–0.47)**	**0.30 (0.19–0.47)**
Abuja and Lagos Nigeria	**2.14 (1.64–2.80)**	**1.53 (1.04–2.27)**
Marital status		
Not married	Ref.	–
Married	0.84 (0.70–1.00)	0.90 (0.75–1.09)
Education		
None or some primary	Ref.	–
Primary or some secondary	**0.80 (0.65–0.99)**	0.84 (0.67–1.05)
Secondary and above	1.24 (0.99–1.54)	0.85 (0.64–1.13)
Employment status		
Unemployed	**0.62 (0.52–0.73)**	1.04 (0.81–1.35)
Employed	Ref.	–
Alcohol use		
No	Ref.	–
Yes	**1.77 (1.41–2.20)**	**1.34 (1.05–1.70)**
Recreational drug use		
No	Ref.	–
Yes	1.65 (0.90–3.03)	0.97 (0.51–1.85)
Enough food to eat^a^		
No	Ref.	–
Yes	0.96 (0.81–1.14)	1.03 (0.84–1.26)
Ever incarcerated		
No	Ref.	–
Yes	**1.48 (1.15–1.92)**	**1.42 (1.07–1.88)**
Distance to clinic		
< 10 km	Ref.	–
10+ km	1.17 (0.98–1.40)	1.16 (0.95–1.40)
Time to clinic		
≤ 30 min	Ref.	–
> 30 min	0.86 (0.73–1.01)	**0.79 (0.67–0.94)**
Waiting time		
Satisfied	Ref.	–
Needs to improve	**1.34 (1.03–1.73)**	1.18 (0.87–1.60)
Depression^b^		
No	Ref.	–
Yes	**1.83 (1.46–2.28)**	**1.47 (1.13–1.91)**
TB diagnosis^c^		
No	Ref.	–
Yes	0.71 (0.28–1.82)	0.60 (0.25–1.43)
Year started ART		
1999–2005	Ref.	–
2006–2010	0.85 (0.45–1.60)	0.80 (0.41–1.55)
2011–2015	1.58 (0.86–2.92)	0.90 (0.46–1.78)
2016–2019	1.39 (0.74–2.64)	0.79 (0.39–1.62)
Duration on ART		
< 2 years	Ref.	–
≥ 2 years to < 4 years	0.85 (0.71–1.02)	0.98 (0.80–1.20)
≥ 4 years	**0.52 (0.43–0.63)**	**0.72 (0.55–0.95)**
Experienced HIV stigma^d^		
No	Ref.	–
Yes	**1.68 (1.17–2.42)**	1.07 (0.72–1.59)
Disclosed HIV status^e^		
No	Ref.	–
Yes	1.09 (0.93–1.28)	1.08 (0.90–1.31)
Missed days of ART in past month		
No days missed	Ref.	–
1–2 days missed	**2.52 (2.03–3.12)**	**2.09 (1.65–2.64)**
3+ days missed	**11.19 (8.86–14.14)**	**7.06 (5.43–9.19)**
CD4 count		
< 200 cells/mm^3^	**1.81 (1.44–2.27)**	1.28 (1.00–1.66)
≥ 200 cells/mm^3^	Ref.	–
Viral load		
< 1000 copies/mL	Ref.	–
≥ 1000 copies/mL	**3.32 (2.72–4.05)**	**2.50 (2.00–3.12)**

Logistic regression with generalized estimating equations, clustered by participant to account for repeated measures, was used to estimate unadjusted and adjusted odds ratios (ORs) and 95% confidence intervals (CIs) for factors potentially associated with missed HIV clinic visits in the previous 6-month period. All potential risk factors specified a priori were included in the adjusted model regardless of significance of the association. We tested for multicollinearity using the variable inflation factor (VIF); all variables included in the adjusted model had a VIF < 2. Analyses were restricted to complete cases only. Bold indicates significance at p < 0.05

^a^Enough food to eat in the past 12 months

^b^Center for Epidemiologic Studies Depression (CES-D) Scale score, dichotomized with a score of 16 or greater suggestive of depression

^c^Tuberculosis (TB) coinfection, diagnosed by a positive GeneXpert, mycobacterial smear or mycobacterial culture result

^d^Participants were defined as experiencing stigma if they had experienced any of the following: social isolation, physical violence, broken family relationships

^e^Disclosure status was defined as disclosure to any of the following individuals: spouse/partner, parent, sibling, children, grandparents, extended family members, friend, roommate, church members

As compared to those who lived 30 min or less away from the clinic, missed clinic visits were less common among those who traveled longer than 30 min to the clinic (aOR 0.79, 95% CI 0.67–0.94). In the unadjusted model, dissatisfaction with clinic waiting times was associated with an increased odds of missing clinic visits (OR 1.34, 95% CI 1.03–1.73); however, this association did not remain significant after adjusting for other factors.

As compared to PLWH who did not use alcohol in the past 6 months, those who used alcohol were more likely to have missed HIV clinic visits in the same 6-month period (aOR 1.34, 95% CI 1.05–1.70). A lifetime history of incarceration was associated with increased odds of missing routine clinic visits (aOR 1.42, 95% CI 1.07–1.88). As compared to those without depression, missing clinic visits was more common among those with a CES-D score suggestive of clinical depression (aOR 1.47, 95% CI 1.13–1.91).

As compared to PLWH who did not miss any days of ART in the past month, missed clinic visits were more common among those who missed 1–2 days of ART (aOR 2.09, 95% CI 1.65–2.64) and 3 or more days of ART (aOR 7.06, 95% CI 5.43–9.19). Missed clinic visits in the past 6 months were also more common among those with a viral load ≥ 1000 copies/mL as compared to those with a viral load < 1000 copies/mL (aOR 2.50, 95% CI 2.00–3.12).

### Reasons for missing visits

**Fig. 3:**
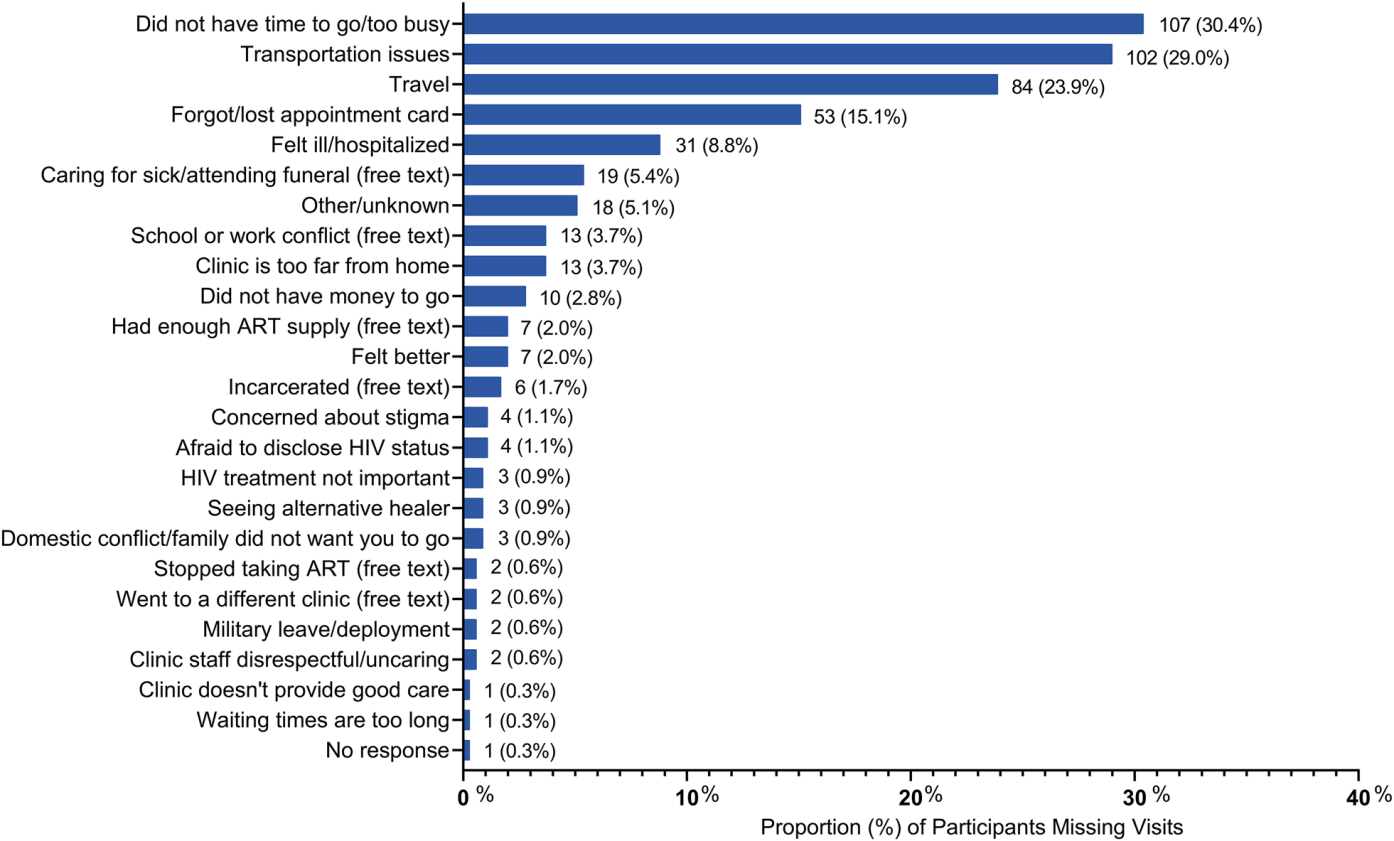
Reasons for not attending HIV clinic visits in the past 6 months. There were 352 unique participants who missed HIV clinic visits in the 6 months prior to any study visit. Participants could provide more than one response and/or specify a free text response. Free text responses were retrospectively recoded into an existing option or coded as a new category. Free text (unsolicited) responses are indicated as such

Among 352 unique participants missing HIV clinic visits in the 6 months prior to any study visit, the top five reasons for missing clinic visits included: did not have time (n = 107, 30.4%), transportation issues (n = 102, 29.0%), travel (n = 84, 23.9%), forgot about the appointment or lost appointment card (n = 53, 15.1%) and felt ill (n = 31, 8.8%) (Fig. 3).

### Sensitivity analyses

The findings of the primary analysis were robust to a sensitivity analysis using multinomial logistic regression to assess factors associated with of the proportion of expected visits missed in the previous 6-months (Additional file 1: Tables S1, S2, S3). Similar to the main analysis, younger age, program site, shorter duration on ART, travel time to clinic, alcohol use, depression, more missed days of ART, and unsuppressed viral load were associated with missed clinic visits in the previous 6-months. Of note, HIV status disclosure and CD4 count < 200 cells/mm^3^ were significantly associated with missing 100% of expected visits as compared to missing 0% of expected visits after adjustment, and history of incarceration was not significantly associated with missing visits, which was dissimilar to the primary analysis.

## Discussion

Missed clinic visits were more common among younger PLWH, those who had been on ART for less time, and those with depression, higher viral load, ART non-adherence, alcohol dependence, and a history of incarceration.

We found that young PLWH and those on ART for less time were more likely to miss routine clinic visits. This finding provides rationale for existing PEPFAR initiatives, including differentiated service delivery and client-centered approaches that allow care to better fit into a client’s life without adding extra burden, thus supporting adherence and engagement. Adolescents and young adults living with HIV have worse treatment adherence and viral suppression and are more likely to be lost to follow-up compared to older adults [25–30]. Furthermore, young people are behaviorally at highest risk for sexually transmitted infections [30, 31]. Strategies that provide quality care with minimal burden for young PLWH is critical.

Although we did not find longer travel times to be a barrier to attending clinic visits nor did we observe any significant associations between clinic waiting time and missing visits, the top two self-reported reasons for missing visits were not having enough time and transportation issues. Clinic accessibility and long clinic waiting times have previously been reported as reasons for intermittent adherence to care [2, 12, 32, 33]. It is important to note that PLWH are not a homogenous group, as evidence suggests that some individuals value the clinic experience and may accept more frequent visits, while others value ease of access and would accept longer waiting times and a rude provider to have less frequent visits [34]. Nonetheless, our findings provide support for client-centered approaches and differentiated service delivery options such as decentralized drug distribution and telemedicine consults that could reduce the burden of accessing care for both clinic clients and health systems, and which may improve engagement across a spectrum of client preferences [16].

Missed clinic visits were more common among those with clinical depression and those reporting alcohol use. A prior assessment of depression in AFRICOS found that 18–25% of PLWH met criteria for clinical depression and that depression was associated with higher viral load independent of ART adherence [35]. Interactions between alcohol use, risk-taking behaviors, ART non-adherence and poor treatment outcomes among PLWH are well established [36–39]. There is precise need for better mental health care including treatment of alcohol dependence to facilitate clinical engagement and overall wellbeing among PLWH. Additionally, we found that a lifetime history of incarceration was associated with missing routine clinic visits. Strategies to identify and support vulnerable prisoners and previously incarcerated individuals should be considered so that potential reincarceration doesn’t lead to intermittent adherence and development of drug resistance.

Consistent with prior studies [1–11], we found that missed visits were associated with higher viral load and ART non-adherence. While we were unable to determine the direction of these associations in these exploratory analyses, these findings suggest interdependence between missed clinic visits, ART non-adherence and viral suppression, and highlight potential drivers of or consequences of missed visits, emphasizing the importance of quality care and engagement for successful treatment outcomes. These associations warrant further investigations to ascertain causal pathways.

This study has several limitations, including that frequency of and adherence to clinic visits was based on self-report, and potentially influenced by social desirability bias. This may result in an underestimation of individuals missing visits or the true number of missed visits. Additionally, we are likely capturing factors associated with clients’ perception of their retention, not with retention itself, which may not be the same. Exclusion from enrollment of certain individuals may have introduced selection bias if these individuals were at higher risk for missing visits. The small proportion reporting missed visits may alternatively reflect that participants enrolled in a clinic-based cohort study are more motivated to stay engaged in care than the general population of PLWH. We were unable to assess whether those who missed study visits were also more likely to miss clinic visits, as data on missed clinic visits is not collected when a study visit is missed. We were also unable to account for history of missed visits which may be associated with future missed visits. We acknowledge that the CES-D scale contains items that are not context-specific for sub-Saharan Africa and therefore complicate interpretation of findings related to depression in our study. As we were not able to determine the direction of the associations observed, it may be reasonable to posit that clinic attendance influences overall health and wellness among PLWH. Finally, we could not adequately explore site level differences that may help to explain important structural, social, or cultural heterogeneity that impact clinic visit adherence. Future studies should interrogate site-specific factors that may be associated with or protective against missing clinic visits.

## Conclusions

Associations with missed clinic visits corroborates existing literature on intermittent clinic adherence and supports the need for strategies targeted at engaging youth and those with depression. Furthermore, our findings provide support for existing client-centered approaches and differentiated service delivery options, as endorsed by the World Health Organization, that simplify HIV services and adapt to the evolving needs of PLWH. Community-based participatory research and PLWH-led interventions are likely crucial for successfully adapting services to specific local contexts.

## Supplementary Information

**Additional file 1.** Additional tables.

## Data Availability

The Henry M. Jackson Foundation for the Advancement of Military Medicine (HJF) and the Water Reed Army Institute of Research (WRAIR) are committed to safeguarding the privacy of research participants. Distribution of data will require compliance with all applicable regulatory and ethical processes, including establishment and approval of an appropriate data-sharing agreement. To request a minimal data set, please contact the data coordinating and analysis center (DCAC) at PubRequest@hivresearch.org and indicate the RV329 study along with the name of the manuscript.

## Abbreviations

AFRICOS: African Cohort Study
AIDS: Acquired immune deficiency syndrome
ART: Antiretroviral therapy
BMI: Body mass index
CES-D: Center for Epidemiological Studies-Depression
CI: Confidence interval
HIV: Human immunodeficiency virus
IQR: Interquartile range
IRB: Institutional review board
OR: Odds ratio
PEPFAR: President’s emergency plan for AIDS relief
PLWH: People living with HIV
TB: Tuberculosis
VIF: Variable inflation factor
WHO: World Health Organization
