# Supplementary material for: Routine HIV clinic visit adherence in the African Cohort Study
Source: AIDS Res Ther. 2022 Jan 7;19:1. doi: 10.1186/s12981-021-00425-0 (PMC8742415; doi:10.1186/s12981-021-00425-0)
Supplement: Supplementary file 1 — Additional file 1. Additional tables. [file 12981_2021_425_MOESM1_ESM.docx]

Supplementary table 1. Characteristics of AFRICOS participants living with HIV on ART attending routine HIV clinic visits at first study visit on antiretroviral therapy, by proportion of expected visits missed

|  | Total  (n=2,771) | 0% missed  (n=2,581) | 1-49% missed  (n=104) | 50-99% missed  (n=64) | 100% missed  (n=22) | p-value |
| --- | --- | --- | --- | --- | --- | --- |
| Age at visit (years) |  |  |  |  |  | *0.18* |
| 18-29 | 528 (19.1%) | 484 (18.8%) | 28 (26.9%) | 10 (15.6%) | 6 (27.3%) |  |
| 30-39 | 979 (35.3%) | 905 (35.1%) | 43 (41.3%) | 25 (39.1%) | 6 (27.3%) |  |
| 40-49 | 788 (28.4%) | 739 (28.6%) | 22 (21.2%) | 19 (29.7%) | 8 (36.4%) |  |
| 50+ | 476 (17.2%) | 453 (17.6%) | 11 (10.6%) | 10 (15.6%) | 2 (9.1%) |  |
| Sex |  |  |  |  |  | *0.10* |
| Male | 1,151 (41.5%) | 1,083 (42.0%) | 31 (29.8%) | 27 (42.2%) | 10 (45.5%) |  |
| Female | 1,620 (58.5%) | 1,498 (58.0%) | 73 (70.2%) | 37 (57.8%) | 12 (54.5%) |  |
| Program site |  |  |  |  |  | ***<0.001*** |
| Kayunga, Uganda | 474 (17.1%) | 424 (16.4%) | 35 (33.7%) | 9 (14.1%) | 6 (27.3%) |  |
| South Rift Valley, Kenya | 987 (35.6%) | 922 (35.7%) | 29 (27.9%) | 25 (39.1%) | 11 (50.0%) |  |
| Kisumu West, Kenya | 498 (18.0%) | 466 (18.1%) | 12 (11.5%) | 17 (26.6%) | 3 (13.6%) |  |
| Mbeya, Tanzania | 520 (18.8%) | 505 (19.6%) | 13 (12.5%) | 1 (1.6%) | 1 (4.5%) |  |
| Abuja & Lagos Nigeria | 292 (10.5%) | 264 (10.2%) | 15 (14.4%) | 12 (18.8%) | 1 (4.5%) |  |
| Marital status |  |  |  |  |  | *0.33* |
| Not married | 1,203 (43.4%) | 1,109 (43.0%) | 52 (50.0%) | 30 (46.9%) | 12 (54.5%) |  |
| Married | 1,568 (56.6%) | 1,472 (57.0%) | 52 (50.0%) | 34 (53.1%) | 10 (45.5%) |  |
| Education |  |  |  |  |  | *0.41* |
| None or some primary | 907 (32.7%) | 836 (32.4%) | 43 (41.3%) | 21 (32.8%) | 7 (31.8%) |  |
| Primary or some secondary | 1,094 (39.5%) | 1,029 (39.9%) | 35 (33.7%) | 24 (37.5%) | 6 (27.3%) |  |
| Secondary and above | 770 (27.8%) | 716 (27.7%) | 26 (25.0%) | 19 (29.7%) | 9 (40.9%) |  |
| Employment status |  |  |  |  |  | ***0.02*** |
| Unemployed | 1,667 (60.2%) | 1,570 (60.8%) | 49 (47.1%) | 33 (51.6%) | 15 (68.2%) |  |
| Employed | 1,104 (39.8%) | 1,011 (39.2%) | 55 (52.9%) | 31 (48.4%) | 7 (31.8%) |  |
| Alcohol use |  |  |  |  |  | ***<0.001*** |
| No | 2,330 (84.1%) | 2,185 (84.7%) | 87 (83.7%) | 42 (65.6%) | 16 (72.7%) |  |
| Yes | 441 (15.9%) | 396 (15.3%) | 17 (16.3%) | 22 (34.4%) | 6 (27.3%) |  |
| Recreational drug use |  |  |  |  |  | *0.64* |
| No | 2,706 (97.7%) | 2,522 (97.7%) | 100 (96.2%) | 63 (98.4%) | 21 (95.5%) |  |
| Yes | 65 (2.3%) | 59 (2.3%) | 4 (3.8%) | 1 (1.6%) | 1 (4.5%) |  |
| Enough food to eat^a^ |  |  |  |  |  | *0.07* |
| No | 934 (33.7%) | 878 (34.0%) | 24 (23.1%) | 26 (40.6%) | 6 (27.3%) |  |
| Yes | 1,837 (66.3%) | 1,703 (66.0%) | 80 (76.9%) | 38 (59.4%) | 16 (72.7%) |  |
| Ever incarcerated |  |  |  |  |  | *0.11* |
| No | 2,477 (89.4%) | 2,317 (89.8%) | 87 (83.7%) | 55 (85.9%) | 18 (81.8%) |  |
| Yes | 294 (10.6%) | 264 (10.2%) | 17 (16.3%) | 9 (14.1%) | 4 (18.2%) |  |
| Distance to clinic |  |  |  |  |  | *0.52* |
| <10 km | 1,439 (51.9%) | 1,333 (51.6%) | 55 (52.9%) | 39 (60.9%) | 12 (54.5%) |  |
| 10+ km | 1,332 (48.1%) | 1,248 (48.4%) | 49 (47.1%) | 25 (39.1%) | 10 (45.5%) |  |
| Time to clinic |  |  |  |  |  | *0.78* |
| <30 minutes | 1,081 (39.0%) | 1,005 (38.9%) | 41 (39.4%) | 28 (43.8%) | 7 (31.8%) |  |
| >30 minutes | 1,690 (61.0%) | 1,576 (61.1%) | 63 (60.6%) | 36 (56.3%) | 15 (68.2%) |  |
| Waiting time |  |  |  |  |  | ***0.04*** |
| Satisfied | 2,356 (85.0%) | 2,200 (85.2%) | 79 (76.0%) | 58 (90.6%) | 19 (86.4%) |  |
| Needs to improve | 415 (15.0%) | 381 (14.8%) | 25 (24.0%) | 6 (9.4%) | 3 (13.6%) |  |
| Depression^b^ |  |  |  |  |  | *0.19* |
| No | 2,405 (86.8%) | 2,246 (87.0%) | 89 (85.6%) | 50 (78.1%) | 20 (90.9%) |  |
| Yes | 366 (13.2%) | 335 (13.0%) | 15 (14.4%) | 14 (21.9%) | 2 (9.1%) |  |
| TB diagnosis^c^ |  |  |  |  |  | *0.80* |
| No | 2,690 (97.1%) | 2,504 (97.0%) | 102 (98.1%) | 63 (98.4%) | 21 (95.5%) |  |
| Yes | 81 (2.9%) | 77 (3.0%) | 2 (1.9%) | 1 (1.6%) | 1 (4.5%) |  |
| Year started ART |  |  |  |  |  | *0.55* |
| 1999-2005 | 95 (3.4%) | 89 (3.4%) | 2 (1.9%) | 3 (4.7%) | 1 (4.5%) |  |
| 2006-2010 | 731 (26.4%) | 683 (26.5%) | 25 (24.0%) | 14 (21.9%) | 9 (40.9%) |  |
| 2011-2015 | 1,326 (47.9%) | 1,227 (47.5%) | 56 (53.8%) | 32 (50.0%) | 11 (50.0%) |  |
| 2016-2019 | 619 (22.3%) | 582 (22.5%) | 21 (20.2%) | 15 (23.4%) | 1 (4.5%) |  |
| Duration on ART |  |  |  |  |  | ***0.03*** |
| <2 years | 1,564 (56.4%) | 1,460 (56.6%) | 65 (62.5%) | 34 (53.1%) | 5 (22.7%) |  |
| >2 years to <4 years | 348 (12.6%) | 318 (12.3%) | 14 (13.5%) | 10 (15.6%) | 6 (27.3%) |  |
| >4 years | 859 (31.0%) | 803 (31.1%) | 25 (24.0%) | 20 (31.3%) | 11 (50.0%) |  |
| Experienced HIV stigma^d^ |  |  |  |  |  | *0.48* |
| No | 2,521 (91.0%) | 2,345 (90.9%) | 96 (92.3%) | 58 (90.6%) | 22 (100.0%) |  |
| Yes | 250 (9.0%) | 236 (9.1%) | 8 (7.7%) | 6 (9.4%) | 0 (0.0%) |  |
| Disclosed HIV status^e^ |  |  |  |  |  | *0.41* |
| No | 450 (16.2%) | 412 (16.0%) | 23 (22.1%) | 11 (17.2%) | 4 (18.2%) |  |
| Yes | 2,321 (83.8%) | 2,169 (84.0%) | 81 (77.9%) | 53 (82.8%) | 18 (81.8%) |  |
| Missed days of ART in past month |  |  |  |  |  | ***<0.001*** |
| No days missed | 2,344 (84.6%) | 2,227 (86.3%) | 68 (65.4%) | 36 (56.3%) | 13 (59.1%) |  |
| 1-2 days missed | 306 (11.0%) | 266 (10.3%) | 21 (20.2%) | 11 (17.2%) | 8 (36.4%) |  |
| 3+ days missed | 121 (4.4%) | 88 (3.4%) | 15 (14.4%) | 17 (26.6%) | 1 (4.5%) |  |
| CD4 count |  |  |  |  |  | *0.26* |
| <200 cells/mm^3^ | 420 (15.2%) | 387 (15.0%) | 21 (20.2%) | 7 (10.9%) | 5 (22.7%) |  |
| >200 cells/mm^3^ | 2,351 (84.8%) | 2,194 (85.0%) | 83 (79.8%) | 57 (89.1%) | 17 (77.3%) |  |
| Viral load |  |  |  |  |  | ***0.001*** |
| <1000 copies/mL | 2,418 (87.3%) | 2,269 (87.9%) | 79 (76.0%) | 52 (81.3%) | 18 (81.8%) |  |
| >1000 copies/mL | 353 (12.7%) | 312 (12.1%) | 25 (24.0%) | 12 (18.8%) | 4 (18.2%) |  |

Between January 2013 and March 2020, people living with HIV were enrolled at 12 HIV clinics in Kenya, Uganda, Tanzania, and Nigeria. Characteristics are summarized from their first study visit on antiretroviral therapy, which may have been at enrollment into the cohort or later if they were ART-naïve at enrollment. Missed HIV clinic visits were based on self-report and measured as the number of clinic visits missed in the past six months. The expected number of clinic visits was based on the self-reported frequency of follow-up visits in a six-month period. The proportion of expected clinic visits missed was calculated by dividing the number of visits missed by the total number of expected clinic visits in the past 6 months and categorized into four levels as follows: 0% missed, 1-49% missed, 50-99% missed, 100% missed. Data are presented as n (column %). P-values were calculated using Pearson’s chi-squared tests and statistically significant p-values (p<0.05) are shown in **bold**.

^a^ Enough food to eat in the past 12 months

^b^ Center for Epidemiologic Studies Depression (CES-D) Scale score, dichotomized with a score of 16 or greater suggestive of depression

^c^ Tuberculosis (TB) coinfection, diagnosed by a positive GeneXpert, mycobacterial smear or mycobacterial culture result

^d^ Participants were defined as experiencing stigma if they had experienced any of the following: social isolation, physical violence, broken family relationships

^e^ Disclosure status was defined as disclosure to any of the following individuals: spouse/partner, parent, sibling, children, grandparents, extended family members, friend, roommate, church members

Supplementary table 2. Unadjusted multinomial logistic regression models comparing factors collected at each 6-monthly study visit with the proportion of expected visits missed in the previous 6-months compared to participants with 0% of expected visits missed

|  | 1-49% missed  Unadjusted OR  (95% CI) | 50-99% missed  Unadjusted OR  (95% CI) | 100% missed  Unadjusted OR  (95% CI) |
| --- | --- | --- | --- |
| Age at visit (years) |  |  |  |
| 18-29 | **4.68 (2.98-7.34)** | **2.30 (1.45-3.65)** | 1.59 (0.71-3.56) |
| 30-39 | **2.57 (1.68-3.92)** | **1.93 (1.31-2.86)** | **1.91 (1.01-3.59)** |
| 40-49 | **1.58 (1.02-2.44)** | 1.45 (0.97-2.15) | 1.44 (0.76-2.71) |
| 50+ | Ref | - |  |
| Sex |  |  |  |
| Male | Ref | - | - |
| Female | 1.19 (0.91-1.57) | 1.29 (0.99-1.68) | 0.74 (0.48-1.16) |
| Program site |  |  |  |
| Kayunga, Uganda | Ref | - | - |
| South Rift Valley, Kenya | **0.22 (0.16-0.31)** | **1.98 (1.29-3.03)** | 1.66 (0.84-3.28) |
| Kisumu West, Kenya | **0.14 (0.09-0.22)** | **2.50 (1.62-3.86)** | 0.42 (0.15-1.19) |
| Mbeya, Tanzania | **0.35 (0.24-0.51)** | **0.18 (0.07-0.46)** | 0.55 (0.19-1.58) |
| Abuja & Lagos Nigeria | **0.66 (0.46-0.94)** | **7.88 (5.09-12.21)** | **5.87 (2.89-11.94)** |
| Marital status |  |  |  |
| Not married | Ref | - | - |
| Married | 0.89 (0.69-1.13) | 0.79 (0.62-1.01) | 1.03 (0.65-1.62) |
| Education |  |  |  |
| None or some primary | Ref | - | - |
| Primary or some secondary | **0.66 (0.49-0.89)** | 0.91 (0.67-1.23) | 0.75 (0.41-1.39) |
| Secondary and above | **0.67 (0.49-0.92)** | **1.67 (1.23-2.27)** | **2.25 (1.32-3.83)** |
| Employment status |  |  |  |
| Unemployed | **0.46 (0.36-0.59)** | **0.69 (0.54-0.89)** | **0.54 (0.34-0.84)** |
| Employed | Ref | - | - |
| Alcohol use |  |  |  |
| No | Ref | - | - |
| Yes | **1.40 (1.01-1.93)** | **2.00 (1.49-2.68)** | **2.74 (1.65-4.56)** |
| Recreational drug use |  |  |  |
| No | Ref | - | - |
| Yes | **1.70 (1.35-5.42)** | 0.68 (0.18-2.59) | **4.69 (1.59-13.79)** |
| Enough food to eat^a^ |  |  |  |
| No | Ref | - | - |
| Yes | 1.29 (0.97-1.70) | 0.89 (0.70-1.15) | 1.18 (0.71-1.99) |
| Ever incarcerated |  |  |  |
| No | Ref | - | - |
| Yes | **1.99 (1.39-2.86)** | 0.95 (0.65-1.38) | 1.60 (0.87-2.93) |
| Distance to clinic |  |  |  |
| <10 km | Ref | - | - |
| 10+ km | 1.03 (0.79-1.32) | 1.24 (0.97-1.58) | **1.73 (1.10-2.71)** |
| Time to clinic |  |  |  |
| <30 minutes | Ref | - | - |
| >30 minutes | 0.83 (0.65-1.06) | 0.84 (0.66-1.07) | 0.91 (0.58-1.44) |
| Waiting time |  |  |  |
| Satisfied | Ref | - | - |
| Needs to improve | **1.67 (1.17-2.39)** | 1.18 (0.80-1.75) | 1.19 (0.50-2.86) |
| Depression^b^ |  |  |  |
| No | Ref | - | - |
| Yes | **2.15 (1.55-2.98)** | **1.84 (1.32-2.56)** | 1.09 (0.47-2.52) |
| TB diagnosis^c^ |  |  |  |
| No | Ref | - | - |
| Yes | 1.30 (0.49-3.47) | 0.29 (0.04-2.07) | 2.62 (0.65-10.59) |
| Year started ART |  |  |  |
| 1999-2005 | Ref | - | - |
| 2006-2010 | 0.77 (0.35-1.72) | 0.73 (0.32-1.65) | 1.22 (0.37-4.06) |
| 2011-2015 | 1.59 (0.73-3.46) | 1.51 (0.69-3.33) | 1.18 (0.37-3.82) |
| 2016-2019 | 1.43 (0.63-3.26) | 1.34 (0.59-3.06) | 1.13 (0.32-3.99) |
| Duration on ART |  |  |  |
| <2 years | Ref | - | - |
| >2 years to <4 years | **0.54 (0.40-0.72)** | 1.24 (0.95-1.62) | 1.42 (0.74-2.73) |
| >4 years | **0.32 (0.25-0.42)** | **0.65 (0.48-0.87)** | 1.22 (0.68-2.19) |
| Experienced HIV stigma^d^ |  |  |  |
| No | Ref | - | - |
| Yes | **2.09 (1.26-3.47)** | **1.73 (1.03-2.92)** | 1.02 (0.25-4.15) |
| Disclosed HIV status^e^ |  |  |  |
| No | Ref | - | - |
| Yes | 0.94 (0.75-1.18) | 0.93 (0.74-1.18) | **2.14 (1.29-3.55)** |
| Missed days of ART in past month |  |  |  |
| No days missed | Ref | - | - |
| 1-2 days missed | **2.97 (2.20-4.00)** | **2.80 (2.11-3.71)** | **3.74 (2.18-6.44)** |
| 3+ days missed | **14.74 (10.91-19.92)** | **11.97 (8.89-16.11)** | **17.53 (10.28-29.91)** |
| CD4 count |  |  |  |
| <200 cells/mm^3^ | **1.88 (1.35-2.61)** | **1.64 (1.14-2.37)** | **3.33 (2.04-5.45)** |
| >200 cells/mm^3^ | Ref | - | - |
| Viral load |  |  |  |
| <1000 copies/mL | Ref | - | - |
| >1000 copies/mL | **4.04 (3.07-5.32)** | **3.44 (2.60-4.55)** | **4.86 (3.03-7.78)** |

We ran a sensitivity analysis using multinomial logistic regression to assess factors associated with the proportion of expected visits missed in the previous six-month period, comparing the following categories of proportions of expected visits missed to participants who missed zero percent of their expected visits: 1-49% missed, 50-99% missed, 100% missed. To account for multiple visits by a participant, analyses were clustered on participant and included a robust variance estimator. Like the main model, all potential predictors were included in the fully adjusted model. **Bold** indicates significance at p<0.05.

^a^ Enough food to eat in the past 12 months

^b^ Center for Epidemiologic Studies Depression (CES-D) Scale score, dichotomized with a score of 16 or greater suggestive of depression

^c^ Tuberculosis (TB) coinfection, diagnosed by a positive GeneXpert, mycobacterial smear or mycobacterial culture result

^d^ Participants were defined as experiencing stigma if they had experienced any of the following: social isolation, physical violence, broken family relationships

^e^ Disclosure status was defined as disclosure to any of the following individuals: spouse/partner, parent, sibling, children, grandparents, extended family members, friend, roommate, church members

Supplementary table 3. Adjusted multinomial logistic regression models comparing factors collected at each 6-monthly study visit with the proportion of expected visits missed in the previous 6-months compared to participants with 0% of expected visits missed

|  | 1-49% missed  Adjusted OR  (95% CI) | 50-99% missed  Adjusted OR  (95% CI) | 100% missed  Adjusted OR  (95% CI) |
| --- | --- | --- | --- |
| Age at visit (years) |  |  |  |
| 18-29 | **2.93 (1.80-4.77)** | **1.81 (1.10-2.98)** | 2.10 (0.90-4.87) |
| 30-39 | **1.79 (1.15-2.77)** | 1.30 (0.86-1.96) | 1.72 (0.90-3.27) |
| 40-49 | 1.36 (0.88-2.08) | 1.28 (0.85-1.91) | 1.44 (0.76-2.71) |
| 50+ | Ref | - | - |
| Sex |  |  |  |
| Male | Ref | - | - |
| Female | 1.22 (0.88-1.68) | 1.26 (0.94-1.70) | 0.95 (0.57-1.57) |
| Program site |  |  |  |
| Kayunga, Uganda | Ref | - | - |
| South Rift Valley, Kenya | **0.23 (0.14-0.37)** | **2.97 (1.69-5.20)** | 1.58 (0.54-4.64) |
| Kisumu West, Kenya | **0.13 (0.07-0.23)** | **3.67 (2.05-6.57)** | 0.54 (0.14-1.99) |
| Mbeya, Tanzania | **0.26 (0.15-0.46)** | **0.17 (0.06-0.79)** | 0.35 (0.09-1.44) |
| Abuja & Lagos Nigeria | **0.46 (0.27-0.77)** | **6.35 (3.49-11.54)** | 2.57 (0.88-7.55) |
| Marital status |  |  |  |
| Not married | Ref | - | - |
| Married | 1.10 (0.83-1.44) | 0.77 (0.59-1.01) | 1.00 (0.60-1.65) |
| Education |  |  |  |
| None or some primary | Ref | - | - |
| Primary or some secondary | 0.76 (0.55-1.04) | 0.93 (0.67-1.28) | 0.72 (0.37-1.38) |
| Secondary and above | 0.78 (0.51-1.20) | 0.81 (0.55-1.20) | 1.03 (0.48-2.21) |
| Employment status |  |  |  |
| Unemployed | 1.28 (0.87-1.89) | 0.90 (0.64-1.28) | 0.88 (0.41-1.86) |
| Employed | Ref | - | - |
| Alcohol use |  |  |  |
| No | Ref | - | - |
| Yes | 0.95 (0.68-1.32) | **1.67 (1.18-2.35)** | 1.60 (0.93-2.76) |
| Recreational drug use |  |  |  |
| No | Ref | - | - |
| Yes | 1.87 (0.92-3.80) | 0.33 (0.07-1.57) | 1.65 (0.58-4.73) |
| Enough food to eat^a^ |  |  |  |
| No | Ref | - | - |
| Yes | 0.98 (0.72-1.32) | 1.15 (0.86-1.53) | 0.89 (0.51-1.56) |
| Ever incarcerated |  |  |  |
| No | Ref | - | - |
| Yes | 1.47 (0.98-2.22) | 1.26 (0.84-1.89) | 1.51 (0.76-3.01) |
| Distance to clinic |  |  |  |
| <10 km | Ref | - | - |
| 10+ km | 1.26 (0.95-1.68) | 1.08 (0.83-1.42) | 1.33 (0.82-2.17) |
| Time to clinic |  |  |  |
| <30 minutes | Ref | - | - |
| >30 minutes | **0.68 (0.52-0.88)** | 0.81 (0.63-1.03) | 0.79 (0.49-1.28) |
| Waiting time |  |  |  |
| Satisfied | Ref | - | - |
| Needs to improve | 1.34 (0.91-1.98) | 1.14 (0.74-1.76) | 1.00 (0.38-2.59) |
| Depression^b^ |  |  |  |
| No | Ref | - | - |
| Yes | **1.64 (1.12-2.39)** | **1.45 (1.00-2.10)** | 1.07 (0.46-2.49) |
| TB diagnosis^c^ |  |  |  |
| No | Ref | - | - |
| Yes | 0.76 (0.22-2.60) | 0.22 (0.03-1.56) | 1.50 (0.47-4.80) |
| Year started ART |  |  |  |
| 1999-2005 | Ref | - | - |
| 2006-2010 | 0.72 (0.31-1.65) | 0.72 (0.30-1.71) | 1.43 (0.39-5.20) |
| 2011-2015 | 0.69 (0.29-1.66) | 0.99 (0.42-2.36) | 1.17 (0.31-4.41) |
| 2016-2019 | 0.42 (0.17-1.06) | 1.15 (0.45-2.90) | 1.74 (0.36-8.30) |
| Duration on ART |  |  |  |
| <2 years | Ref | - | - |
| >2 years to <4 years | **0.65 (0.47-0.88)** | 1.33 (0.99-1.77) | 1.79 (0.89-3.59) |
| >4 years | **0.46 (0.30-0.70)** | 0.95 (0.65-1.39) | 1.74 (0.79-3.79) |
| Experienced HIV stigma^d^ |  |  |  |
| No | Ref | - | - |
| Yes | 1.08 (0.63-1.85) | 1.12 (0.63-2.00) | 0.66 (0.15-2.84) |
| Disclosed HIV status^e^ |  |  |  |
| No | Ref | - | - |
| Yes | 1.06 (0.81-1.40) | 0.99 (0.76-1.30) | **1.75 (1.00-3.06)** |
| Missed days of ART in past month |  |  |  |
| No days missed | Ref | - | - |
| 1-2 days missed | **1.99 (1.45-2.73)** | **2.33 (1.69-3.21)** | **3.14 (1.64-6.01)** |
| 3+ days missed | **7.62 (5.38-10.80)** | **7.18 (4.97-10.39)** | **8.69 (4.54-16.63)** |
| CD4 count |  |  |  |
| <200 cells/mm^3^ | 1.25 (0.89-1.76) | 1.13 (0.75-1.73) | **2.12 (1.23-3.68)** |
| >200 cells/mm^3^ | Ref | - | - |
| Viral load |  |  |  |
| <1000 copies/mL | Ref | - | - |
| >1000 copies/mL | **3.14 (2.30-1.76)** | **2.40 (1.74-1.73)** | **2.33 (1.39-3.92)** |

We ran a sensitivity analysis using multinomial logistic regression to assess factors associated with the proportion of expected visits missed in the previous six-month period, comparing the following categories of proportions of expected visits missed to participants who missed zero percent of their expected visits: 1-49% missed, 50-99% missed, 100% missed. To account for multiple visits by a participant, analyses were clustered on participant and included a robust variance estimator. Like the main model, all potential predictors were included in the fully adjusted model. **Bold** indicates significance at p<0.05.

^a^ Enough food to eat in the past 12 months

^b^ Center for Epidemiologic Studies Depression (CES-D) Scale score, dichotomized with a score of 16 or greater suggestive of depression

^c^ Tuberculosis (TB) coinfection, diagnosed by a positive GeneXpert, mycobacterial smear or mycobacterial culture result

^d^ Participants were defined as experiencing stigma if they had experienced any of the following: social isolation, physical violence, broken family relationships

^e^ Disclosure status was defined as disclosure to any of the following individuals: spouse/partner, parent, sibling, children, grandparents, extended family members, friend, roommate, church members
